# Multitarget, multiagent PLGA nanoparticles for simultaneous tumor eradication and TME remodeling in a melanoma mouse model

**DOI:** 10.1007/s13346-023-01413-9

**Published:** 2023-08-23

**Authors:** Asmaa Ramzy, Aya H. Soliman, Sally I. Hassanein, Aya A. Sebak

**Affiliations:** 1 Department of Pharmaceutical Technology, Faculty of Pharmacy & Biotechnology, the German University in Cairo, New Cairo, 11511 Egypt; 2 Department of Pharmaceutical Biology, Faculty of Pharmacy & Biotechnology, the German University in Cairo, New Cairo, 11511 Egypt; 3 Department of Biochemistry, Faculty of Pharmacy & Biotechnology, the German University in Cairo, New Cairo, 11511 Egypt

**Keywords:** Biomaterials, Multi-functional nanoparticles, Immunotherapy, Immunogenic cell death, Tumor microenvironment remodeling, TAMs, TAFs, Combination therapy

## Abstract

Despite the fact that chemoimmunotherapy has emerged as a key component in the era of cancer immunotherapy, it is challenged by the complex tumor microenvironment (TME) that is jam-packed with cellular and non-cellular immunosuppressive components. The aim of this study was to design a nanoparticulate system capable of sufficiently accumulating in the tumor and spleen to mediate local and systemic immune responses, respectively. The study also aimed to remodel the immunosuppressive TME. For such reasons, multi-functional polylactic-co-glycolic acid (PLGA) nanoparticles (NPs) were engineered to simultaneously eradicate the cancer cells, silence the tumor-associated fibroblasts (TAFs), and re-educate the tumor-associated macrophages (TAMs) using doxorubicin, losartan, and metformin, respectively. These agents were also selected for their ability to tip the balance of the splenic immune cells towards immunostimulatory phenotypes. To establish TAM and TAF cultures, normal macrophages and fibroblasts were incubated with B16F10 melanoma cell (Mel)-derived secretome. Drug-loaded PLGA NPs were prepared, characterized, and tested in the target cell types. Organ distribution of fluorescein-loaded PLGA NPs was evaluated in a mouse model of melanoma. Finally, the local and systemic effects of different combination therapy programs were portrayed. The in vitro studies showed that the drug-loaded PLGA NPs could significantly ablate the immunosuppressive nature of Mel and skew TAMs and TAFs towards more favorable phenotypes. While in vivo, PLGA NPs were proven to exhibit long blood circulation time and to localize preferentially in the tumor and the spleen. The combination of either metformin or losartan with doxorubicin was superior to the monotherapy, both locally and systemically. However, the three-agent combo produced detrimental effects in the form of compromised well-being, immune depletion, and metastasis. These findings indicate the potential of TME remodeling as means to prime the tumors for successful chemoimmunotherapy. In addition, they shed light on the importance of the careful use of combination therapies and the necessity of employing dose-reduction strategies.

**Graphical Abstract:**

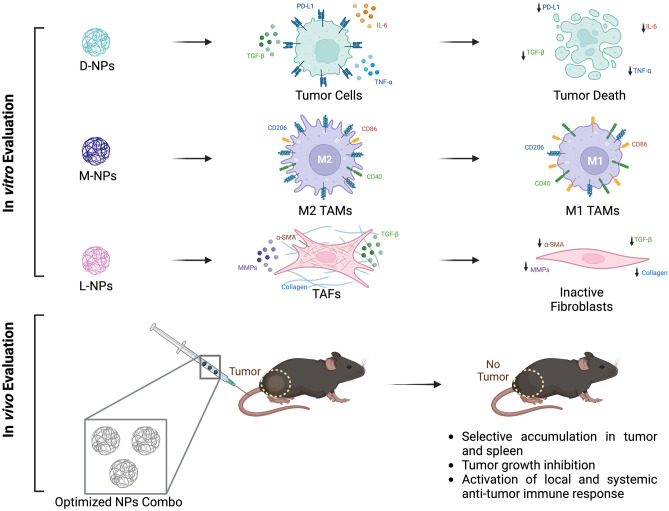

D-NPs doxorubicin-loaded NPs, M-NPs metformin-loaded NPs, L-NPs losartan-loaded NPs, TAMs tumor-associated macrophages, TAFs tumor-associated fibroblasts, PD-L1 programmed death ligand 1, TNF-α tumor necrosis factor alpha, TGF-β transforming growth factor beta, CD206/40/86 cluster of differentiation 206/40/86, α-SMA alpha-smooth muscle actin, MMPs matrix metalloproteases

**Supplementary Information:**

The online version contains supplementary material available at 10.1007/s13346-023-01413-9.

## Introduction

Skin melanoma is an aggressive and highly complex solid tumor. This increases the incidence and limits the therapeutic options especially at advanced stages [1, 2]. However, melanoma immunotherapy has witnessed remarkable progress, and melanoma was among the first malignancies in which immune checkpoint inhibitors (ICIs) were applied [3]. Immunotherapy is, however, not limited to ICIs, whereas accumulating clinical data indicates that the activation of the immune response against dying cancer cells by certain chemotherapeutics, such as doxorubicin, is associated with improved disease outcome as well [4]. This phenomenon is termed immunogenic cell death (ICD) and is a phenomenon in which dying cells undergo changes that recruit antigen-presenting cells (APCs) to sites of active ICD, eventually resulting in the priming of an immune response. Despite the fact that immunotherapy can elicit significant durable overall survival compared to conventional therapy, less than one-third of the patients achieve satisfactory benefits [5]. The complex tumor microenvironment (TME) is what hinders adequate treatment response. The TME comprises tumor cells as well as the surrounding vasculature, extracellular matrix (ECM), several non-tumoral cellular subsets, and signaling molecules [6]. An immunosuppressive microenvironment is created through interactions between different cells of the TME that is crucial to both the development and the progression of cancer. The non-malignant cells of the TME often assume pro-tumorigenic phenotypes that play essential roles in all phases of carcinogenesis [7, 8]. Normal fibroblasts, for instance, evolve into tumor-associated fibroblasts (TAFs) which regulate multiple functions during tumorigenesis. TAFs stimulate tumor cell proliferation and contribute to deposition of ECM components (e.g., collagen and fibronectin) and secretion of tumor-associated proteins and growth factors (e.g., transforming growth factor beta (TGF-β), vascular endothelial growth factor (VEGF), and matrix metalloproteases (MMPs)). TAFs also express specific proteins (e.g., Wnt16) which contribute to drug resistance in malignancies [9, 10]. Tumor-associated macrophages (TAMs), the major immune component in the TME of melanoma, often assume a pro-tumorigenic M2 phenotype. TAMs promote tumorigenesis by secreting growth factors and facilitating matrix remodeling, angiogenesis and metastasis. TAMs have also been shown to suppress the adaptive immunity and have been statistically correlated to poor prognosis and poor patient outcome to different therapies in human and murine malignancies [11, 12].

The lack of adequate concentrations of the therapeutic agents at the target site(s), upon systemic administration, as well as the exposure to the drug efflux mechanisms, represents other contributing factors to the inefficient outcomes of chemoimmunotherapy. Another shortcoming of the systemic administration of the free drug molecules is the deliberate accumulation at non-target sites which brings about the side effects of cardiotoxicity and myelosuppression in the case of doxorubicin [13]. The local application of therapeutic agents remains a valid alternative; however, it is not without drawbacks. Inaccessibility of some tumors, presence of secondary metastatic sites, as well as rapid diffusion of drugs represent some of the drawbacks of localized therapy [14].

Therefore, to maximize the tumor accumulation and minimize the side effects of therapeutic agents, utilization of a tailored drug delivery system is indispensable. In addition, long residence time of the delivery system and the interaction of the payloads with the spleen are pressing needs to guarantee the initiation of an adequate systemic immune response. Among diverse delivery systems, polylactic-co-glycolic acid (PLGA)-based nanoparticles (NPs) represent a promising option for the required properties. In our previous work, PLGA NPs were shown to exhibit versatile physicochemical properties including high entrapment efficiency of the loaded drugs [1]. Moreover, carefully engineered PLGA NPs were shown to preferentially accumulate in the tumor tissue benefiting from the enhanced permeability and retention (EPR) effect. They were also found to favorably recruit tumor-favoring protein corona achieving what is known as endogenous targeting [15, 16]. In addition, PLGA NPs were shown to be immune-inert, a property that is desirable in this study to avoid uncontrollable interaction with the immune organs [17].

Therefore, in order to obtain an adequate therapeutic outcome of chemoimmunotherapy, a synergistic TME-targeted strategy was introduced through a PLGA-based delivery system. In this context, modulation of TAFs and TAMs as the key players contributing to the failure of activation of the anti-tumor immune response was attempted using repurposed drugs. Losartan, an antihypertensive agent, was selected for modulation of TAFs. It exhibits anti-fibrotic properties which could reprogram TAFs and mediate downregulation of different pro-tumorigenic molecules [18]. Metformin, an antihyperglycemic drug, is another agent with a high potential to skew TAMs into the M1 anti-tumorigenic phenotype [19]. Both agents have been associated with favorable clinical outcomes. Losartan was shown to prolong the overall survival for ovarian [20] and prostate [21] cancer patients, when combined with chemotherapy. It was also associated with lower lymph node metastasis in advanced stage lung cancer patients [22] and could also increase the efficacy of adjuvant chemotherapy after tumor resection in patients with cholangiocarcinoma (CCA) [23]. Metformin was associated with beneficial clinical outcomes in patients with advanced melanoma enrolled into immune checkpoint blockade treatment programs [24, 25]. Doxorubicin, losartan, and metformin have as well been shown to modulate the activation and proliferation of the stimulatory immune cells belonging to both the innate and the adaptive immune systems and to suppress the regulatory immune cells in the spleen [26–28]. Therefore, a comprehensive evaluation of the efficacy of the mono- and combination therapies of these agents will be implemented.

## Materials and methods

### Materials

PLGA of lactide:glycolide ratio of 50:50 and molecular weight of 30,000–60,000 Da and partially hydrolyzed polyvinyl alcohol (PVA) of molecular weight of 30,000 Da were purchased from Sigma Aldrich, Germany. Losartan potassium ≥ 99% (HPLC), metformin hydrochloride ≥ 99% (HPLC) and doxorubicin hydrochloride ≥ 99% (HPLC) were purchased form Selleckchem, USA. Murine melanoma (B16F10; CRL-6475), fibroblasts (L929; CCL-1), and macrophages (RAW 264.7; TIB-71) cell lines were purchased from the American Type Culture Collection (ATCC, VA, USA).

### Formulation and characterization

For the preparation of PLGA NPs, a modified water-in-oil-in-water (w/o/w) double emulsion solvent evaporation method was employed [1, 15]. Fifty milligrams of PLGA were dissolved in ethyl acetate at a concentration of 25 mg/mL representing *solution A*. An amount of 5 mg of losartan, metformin, or doxorubicin was dissolved in 1 mL of an aqueous solution of 0.5% PVA representing *solution B* to produce L-NPs, M-NPs, or D-NPs respectively. *Solution B* was then slowly injected into *solution A* and homogenized for 1 min using high-speed homogenization (30,000 rpm) to form a w/o primary emulsion. This was further injected into 10 mL aqueous solution of 0.5% PVA and homogenized for 5 min to form the double emulsion (w/o/w). An amount of 1 mg of sodium fluorescein was used in place of the drugs to prepare f-NPs or concomitantly with metformin or losartan to form f-M-NPs or f-L-NPs, respectively. Plain NPs (P-NPs) were similarly prepared in the absence of drugs or fluorescein. For solvent evaporation, the double emulsion was stirred in an open vessel for 2 h at room temperature under dark conditions. The resulting suspension was then transferred into Amicon Ultra-15 Centrifugal Filter Unit (50 kDa MWCO, Merck Millipore, Germany) and centrifuged at 10,000 × g for 10 min at 4 °C. The flow-through was collected for the determination of the concentration of the unentrapped fraction of the drugs, and the NPs were washed twice using ultrapure water. The NPs were then resuspended in phosphate-buffered saline (PBS), pH 7.4, to a concentration of 3 mg/mL of the loaded drugs and sterile filtered using Merck Millex^™^-GV Sterile Syringe Filter Unit, PVDF, 0.22 µm. This stock suspension of the NPs was then stored at 4 °C until further use. It is worth mentioning that the storage stability of the formulated NPs was not addressed in this study; therefore, NPs were only used within 1 week of fabrication.

Particle size and surface charge were characterized by dynamic light scattering technique using Zetasizer (Malvern, Nano-ZS) at a wavelength of 633 nm of He-Ne laser source. Samples were diluted 10 times in ultrapure water before the analysis [1].

Surface morphology and particle shape were assessed by scanning electron microscopy (SEM) (SUPRA 55, Zeiss, Germany). Samples were diluted 10 times in ultrapure water prior to the imaging procedure. A volume of 50 µL of the diluted suspension was then air-dried on an aluminum SEM stub [29]. Coating by gold sputtering was then performed at a current intensity of 20 mA in Hummer 8.0 sputtering system (Anatech, USA) for 3 min.

Entrapment efficiency % (EE%) of doxorubicin, losartan, or metformin was determined spectrophotometrically at wavelengths 480 nm [30], 210 nm [31], and 231 nm [32] respectively by the indirect method according to Eq. 1.

1 $$\mathrm{EE}\%=\frac{\mathrm{Total}\;\mathrm{drug}\;(\mathrm{mg})-\mathrm{unentrapped}\;\mathrm{drug}\;(\mathrm{mg})}{\mathrm{Total}\;\mathrm{drug}\;(\mathrm{mg})}\times100$$

The flow-through of the NP suspension was diluted in PBS prior to the quantification. Calibration curves and limits-of-detection (LODs) are shown in the supplementary materials (Fig. S1 A–C).

### Cell culture and in vitro assays

RAW 264.7 macrophages (MΦ) or B16F10 melanoma cells (Mel) were cultured in DMEM, while L929 fibroblasts (Fbls) were cultured in RPMI, supplemented with 10% fetal bovine serum (FBS) and 1% penicillin/streptomycin (complete culture medium). TAMs and TAFs were established from MΦ or Fbls respectively by culturing in 50% of Mel-conditioned medium (medium containing Mel secretome) [33] in fresh complete culture media for 48 h. Stromal and immune cell conditioned medium-treated melanoma cells (c-Mel) were obtained by culturing Mel in 25% TAF-conditioned (containing TAF secretome) [34] and 25% TAM-conditioned (containing TAM secretome) [12] media in fresh complete culture medium for 48 h respectively. The conditioned media were obtained and pooled from confluent cultures, sterile filtered using Merck Millex^™^-GV Sterile Syringe Filter Unit, PVDF, 0.22 µm, and stored at −20 °C until further use as previously reported [35, 36].

For the evaluation of the impact of M-NPs or L-NPs on the viability of TAMs or TAFs respectively, 5000 cells/well were seeded in 96-well plates in complete culture medium overnight. Cells were then conditioned with the relevant medium for 48 h. Afterwards, cells were treated with different concentrations of M-NPs or L-NPs respectively, diluted from the stock suspension of NPs (3 mg/mL of metformin or losartan, respectively) in the relevant medium for 24 h, followed by evaluation of the cytotoxicity by MTT assay. Likewise, Mel were seeded at a density of 1000 cells/well for the evaluation of the impact of D-NPs on cell viability. The half maximal inhibitory concentration (IC_50_) was determined by nonlinear regression analysis using GraphPad Prism software 8.3 and was used in each case for molecular analyses.

It was also interesting to evaluate the impact of D-NPs on Mel treated with conditioned medium derived from TAMs treated with IC_50_ of M-NPs and TAFs treated with IC_50_ of L-NPs (tc-Mel) as means to evaluate whether priming TAMs or TAFs would increase Mel sensitivity to the anti-cancer treatment or not. The conditioned media of treated TAFs and TAMs were pre-centrifuged at 20,000 × g for 10 min prior to filtration to eliminate any remnants of the L-NPs and M-NPs respectively.

For the evaluation of the intracellular uptake of the NPs in each cell type, a non-toxic concentration (100 µg/mL, calculated based on the yield % shown in Table S1) of the fluorescein-loaded NPs (f-NPs) was used in place of the drug-loaded NPs. The concentration of the intracellular NPs was determined by measuring the fluorescence intensity of the test samples using a multi-mode microplate reader (Victor 3 V 1420, Perkin Elmer, USA) at excitation/emission wavelengths of 485 nm/535 nm and compared to a calibration curve obtained by measuring the fluorescence intensity of a serial dilution of f-NPs added to the culture of the relevant cell lines [37].

### Melanoma mouse model

Male C57 black 6 (C57/BL6) mice of 20–25 g body weight and age of 8–10 weeks were obtained from the local animal breeds at the German University in Cairo (GUC), Egypt. The experiment protocol adhered to the ARRIVE guidelines and was approved by the Research Ethics Committee of the Faculty of Pharmacy and Biotechnology, GUC (Project ID: PTC-2021-02). Free movement was allowed by housing every 3 mice in appropriately sized cages with standard housing conditions of a 12-h light/dark cycle and a temperature of 22 °C. All mice were daily cared for, received a standard laboratory diet, and were granted free access to water. Animal remains were disposed by incineration according to the approved animal waste disposal system.

Melanoma mouse model was established through the intradermal transplantation of B16F10 cells at a density of 0.5 × 10^6 ^cells in 50 µL of complete culture medium per mouse. External tumor volume was monitored 2–3 times per week until it reached a volume of 100–130 mm^3^. Tumor volume was calculated as shown in Eq. 2.

2 $$\mathrm{Tumor}\;\mathrm{Volume}\;\left(\mathrm{mm}^3\right)=\mathrm{Length}\;\left(\mathrm{mm}\right)\times\mathrm{width}^2\;\left(\mathrm{mm}^2\right)\times0.5236$$

After reaching the target tumor volume, treatment was initiated [16].

### Biodistribution

Mice were randomly grouped into 16 groups (*n* = 3) upon reaching the target tumor volume (100–130 mm^3^) and were then intravenously (IV) injected with 100 µl of saline, f-NPs, f-M-NPs, or f-L-NPs at a concentration of 30 mg/mL (calculated based on the yield % shown in Table S1) in saline once daily. This volume of f-M-NPs and f-L-NPs contains approximately 0.25 mg of metformin or losartan respectively (equivalent to 10 mg/kg of either drug). This dose of metformin or losartan was selected based on the lowest available dose in literature, to the best of our knowledge, that could exert an effect [38, 39]. In addition, it was necessary to ensure that these doses do not impact the tumor growth since metformin and losartan were utilized for their TME and immune system modulatory effects, not as mere anti-cancer agents. For such a reason, the tumor volume was evaluated for mice (*n* = 3) injected with 100 µL of saline, f-NPs, f-M-NPs, or f-L-NPs at the same concentration over 21 days post-transplantation (Fig. S3).

Mice were euthanized by cervical dislocation after predefined time points (1, 3, 5, or 7 days) under light anesthesia. Blood samples (0.5 mL) were withdrawn by cardiac puncture into heparinized vials, and organs/tissues (tumor, spleen, and kidneys) were collected and washed with saline solution. Plasma was separated from blood cells by centrifugation at 1500 × g for 5 min at 4 °C, and all organs were stored in Eppendorf tubes at −80 °C until further use. Organs were then thawed and homogenized in a manual tissue homogenizer using one molar NaOH to achieve a concentration of 0.1 g of tissue per 1 mL of NaOH. Fluorescence intensity was measured using a multi-mode microplate reader (Victor 3 V 1420, Perkin Elmer, USA) at excitation/emission wavelength of 485 nm/535 nm upon dilution of homogenates to 0.01 g/mL. To account for auto-fluorescence, calibration curves were constructed for each of the NPs in homogenates of organs or tissues or in plasma of control mice (0.01 g/mL).

To account for the differences in organ weights in the treated or control animals, amounts of NPs in the organ or tissue homogenates or in plasma obtained from treated or control animals were normalized against organ weight before subtraction of values in control mice from values in treated mice. Biodistribution was expressed as the percentage of injected dose per gram of tissue (%ID/g) at each time point. Specific gravity of 1.021 g/mL was utilized for conversion of plasma volume into mass [40]. Further details on the calculations are available in the Supplementary Materials, Sect. 1.4.

### In vivo efficacy of D-NPs used in monotherapy or in combination with M-NPs and/or L-NPs

After reaching the target tumor volume of 100–130 mm^3^, mice were randomly grouped into five groups and one group of non-tumor-bearing mice served as a normal control (NC) as shown in Table 1.

**Table 1:** Description of the animal groups used in the evaluation of the in vivo efficacy of D-NPs used in monotherapy or in combination with M-NPs and/or L-NPs (*n* = 6)

**Group**	**Group code**	**MEL**	**D-NPs**	**M-NPs**	**L-NPs**
**Normal control**	NC	-	-	-	-
**Tumor control**	TC	+	-	-	-
**Treated group 1**	TG-D	+	+	-	-
**Treated group 2**	TG-DM	+	+	+	-
**Treated group 3**	TG-DL	+	+	-	+
**Treated group 4**	TG-DML	+	+	+	+

*NC* normal control, *TC* tumor control, *TG-D* treated group with D-NPs, *TG-DM* treated group with D-NPs and M-NPs, *TG-DL* treated group with D-NPs and L-NPs, *TG-DML* treated group with D-NPs, M-NPs and L-NPs

D-NPs were applied three times over a course of treatment of 14 days in cycles of 5 days at a dose equivalent to 2 mg/kg doxorubicin per mouse per cycle. M-NPs and L-NPs were applied on a daily basis at a dose equivalent to 10 mg/kg of metformin and losartan respectively. Doses were determined based on previously published data with slight modifications [38, 39, 41]. In the treated groups (TGs) 2–4, NPs loaded with different drugs were mixed prior to administration, and the total volume of injected doses did not exceed 200 µL per mouse per day. Mice in the NC and TC groups received IV saline.

Tumor volume was monitored 2–3 times per week over the course of treatment. Tumor growth rates in TC and the TGs were estimated via calculation of the tumor volume doubling time (DT). Since tumor volume was previously reported to follow exponential growth [42], DT was calculated via nonlinear fit of growth curves “Exponential (Malthusian) growth” using GraphPad Prism software 8.3. At the end of the treatment period, mice were euthanized by cervical dislocation under mild anesthesia.

### Spleen index determination

After the mice were euthanized, the spleens were collected and weighed, and the spleen index was calculated according to Eq. 3 as an indicator of immune system function [43].

3 $$\mathrm{Spleen}\;\mathrm{index}=\mathrm{weight}\;\mathrm{of}\;\mathrm{spleen}\;\left(\mathrm{mg}\right)/\mathrm{weight}\;\mathrm{of}\;\mathrm{mouse}\;\left(\mathrm g\right)$$

### Histopathological analysis

Tissue biopsies were fixed in 10% buffered formalin for 24 h after collection for the histopathological analysis of the skin and spleen of NC, TC, and TGs. Specimens were cleared in xylene and embedded in paraffin at 56 °C in a hot air oven for 24 h. A sledge microtome was used to prepare the paraffin-embedded tissue blocks for sectioning at 4 µm thickness. The tissue sections were stained by Hematoxylin & Eosin (H & E) stains for histopathological examination under a light microscope [44].

### Molecular analyses

#### Intracellular and soluble protein expression analysis by enzyme-linked immunosorbent assay (ELISA)

Supernatants obtained from cell cultures treated with IC_50_ conditions of the respective NPs were treated according to the manufacturer’s protocols for the assessment of the expression of different extracellular proteins and soluble mediators by ELISA. The following mouse kits from MyBiosourse, USA, were utilized: collagen1 (MBS724458), TGF-β (MBS160136), MMP2 (MBS824667), MMP9 (MBS175917), IL-6 (MBS824703), and TNF-α (MBS825075).

Mouse alpha-smooth muscle actin (α-SMA) ELISA kit (MBS267551, MyBioSource, USA) was also used to determine the expression level of the intracellular α-SMA in Fbls and TAFs according to the manufacturer’s protocol.

#### Gene expression analysis by RT-qPCR

A TRIzol RNA extraction reagent (Applied Biosystems, USA) was utilized for the isolation of total RNA. Reverse transcription and relative expression quantification of TNF-α, IL-10, iNOS, Arginase-1, PD-L1, and β-actin mRNAs were performed (TaqMan assay IDs: Mm00443258_m1, Mm01288386_m1, Mm00440502_m1, Mm00475988_m1, Mm01208504_m1, and Mm00607939_s1 respectively). The experimental setup allowed for simultaneous quantification of both target and housekeeping genes in the same PCR tube as probes used for TNF-α, IL-10, iNOS, Arginase-1, and PD-L1 was labeled with the FAM reporter dye, while probes used for β-actin were labeled with the VIC reporter dye. High-Capacity cDNA Reverse Transcription Kit (Applied Biosystems, USA) was used for the reverse transcription process according to the manufacturer’s instructions. This work employed the TaqMan Real-Time q-PCR-StepOneTM Systems (Applied biosystems, USA) for the relative expression analysis of all the targets and housekeeping genes (β–actin). Relative expressions were calculated using the 2^−∆∆CT^ method [45].

#### Cell-surface protein expression analysis by flow cytometry

A modified protocol by Bommareddy et al. has been implemented [46]. In brief, cells were collected by scraping after treatment with the IC_50_ conditions of the respective NPs and washed twice with ice-cold PBS. Zombie Violet^™^ Fixable Viability Kit (423113, Biolgened, USA) was utilized to distinguish viable from dead cells. Staining was performed for 10 min using a 1:500 dilution in PBS under dark conditions. Cells were then washed twice with flow cytometry staining buffer (2% v/v BSA, 2 mM EDTA, 2 mM NaN_3_ in PBS) followed by staining for 40 min with a mixture of the required antibodies (PE anti-mouse CD206 (141706, Biolgened), APC/Cyanine7 anti-mouse CD86 (105030, Biolegend) and PerCP-eFluor™ 710 anti-mouse CD40 (46-0401-82, eBioscience^™^ Invitrogen) antibodies) and TruStain fcX-CD16/32 to block non-specific binding of immunoglobulin to the Fc receptors (101319, Biolegend) at a dilution 1:100 in the same buffer. Following the staining, cells were washed with the buffer twice and finally suspended in 100 µL of the buffer before sample acquisition by Novocyte Flow Cytometer (Acea Biosciences, USA). UltraComp eBeads (01-2222-41, eBioscience^™^ Invitrogen) were utilized for the compensation of the corresponding staining. FlowJo software was used for data analysis, and gate placement was guided using Fluorescence-Minus-One (FMO) controls.

### Statistical analysis

Data was collected from results of at least three replicates of each experiment. Statistical analysis was performed using GraphPad Prism software 8.3 via ordinary one-way, two-way ANOVA, or *t*-test analyses whenever appropriate.

## Results

### PLGA NPs exhibited uniform size negative surface charge and high payload of different drugs

**Fig. 1:**
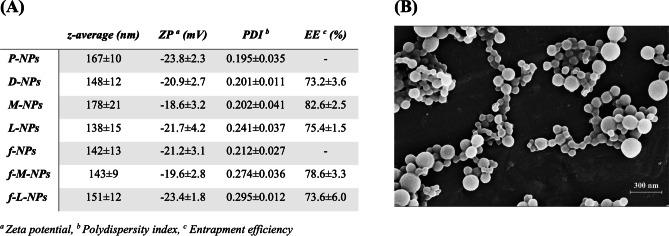
Physicochemical properties of NPs. **A** Particle size (expressed as z-average in nm), surface charge (expressed as zeta potential in mV) and PDI obtained by Malvern Zetasizer and entrapment efficiency % (EE%) obtained by UV-Vis Spectrophotometry. **B** Surface morphology of P-NPs obtained by SEM. P-NPs plain NPs, D-NPs doxorubicin-loaded NPs, M-NPs metformin-loaded NPs, L-NPs losartan-loaded NPs, f-NPs fluorescein-loaded NPs, f-M-NPs fluorescein and metformin co-loaded NPs, f-L-NPs fluorescein and losartan co-loaded NPs

Plain PLGA NPs exhibited an average particle size of 170 nm, an average zeta potential of approximately −20 mV, and a polydispersity index (PDI) less than 0.3 (Fig. 1A). The inclusion of either a drug or a fluorescent dye in the NPs did not impact the particle size or the surface charge. Yet, a high EE% was obtained. Upon comparing L-NPs and M-NPs to their fluorescein co-loaded counterparts (f-L-NPs and f-M-NPs respectively), a slight decrease in EE% was observed. Upon examining the NPs using SEM, they were shown to exhibit a nanometric size distribution, a spherical shape and smooth surface morphology (Fig. 1B).

### In vitro efficiency of NPs in target cell types

#### MΦ exposure to Mel secretome altered their phenotype and the response to M-NPs

**Fig. 2:**
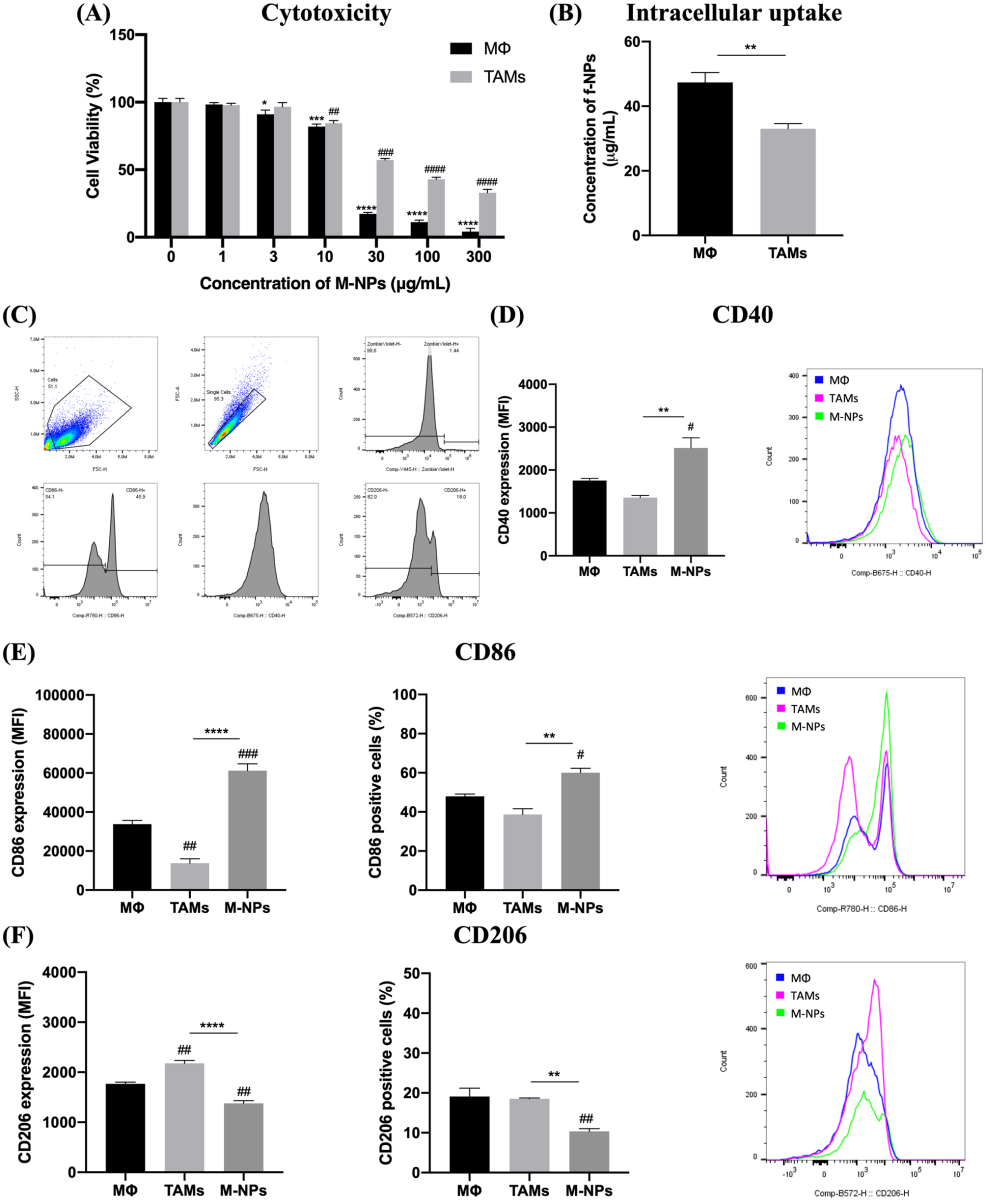
Impact of M-NPs on tumor-associated macrophages (TAMs). **A** Cytotoxicity of M-NPs in normal MΦ and TAMs. **B** Intracellular uptake of f-NPs in normal MΦ and TAMs. **C** The gating strategy employed in the multi-parameter flow cytometry analysis of surface markers of normal MΦ, TAMs, and TAMs treated with IC_50_ (67.1 µg/mL) of M-NPs. The upper left panel indicates selection of the desired cell population based on the forward scatter-height (FSC-H) vs side scatter-height (SSC-H) plot. In the upper middle panel, FSC-H vs FSC-area (FSC-A) plot was utilized to exclude doublet cells. In the upper right panel, zombie violet positive population (dead cells) was excluded. The three lower panels indicate the single parameter histograms of CD86, CD40, and CD206 (from left to right) respectively. An FMO strategy was employed to define positive populations of each surface marker. **D**–**F** Surface expression of CD40, CD86, and CD206 respectively. Median fluorescence intensity (MFI) was utilized to define the relative expression of all markers. Percentage of positive cells in the case of CD86 and CD206 was also indicated. An overlay of representative single-parameter histogram of all markers is inserted. Statistical analyses were performed by *t*-test or one-way ANOVA using GraphPad Prism Software 8.3. In insert **A**, means of treated cells were normalized to the mean of control cells. * denotes comparison in the case of MΦ, and # denotes comparison in the case of TAMs. In inserts **D**–**F**, * denotes comparison to TAMs, and # denotes comparison to MΦ. Levels of significance are indicated as follows: */#*P* < 0.05; **/##*P* < 0.01; ***/###*P* < 0.001; ****/####*P* < 0.0001

The exposure of normal MΦ to Mel secretome not only affected how the cells responded to M-NPs in terms of intracellular uptake and cytotoxicity but also altered the phenotype of MΦ. Onwards, Mel secretome-exposed MΦ are referred to as TAMs. M-NPs were found to be less toxic to TAMs than normal MΦ with an IC_50_ of 67.1 ± 8.6 µg/mL compared to 17.5 ± 2.4 µg/mL respectively (Fig. 2A). The intracellular uptake of f-NPs was also less efficient in TAMs (Fig. 2B). For the evaluation of the phenotype of MΦ, the expression of CD40 and CD86 as markers of M1 phenotype and CD206 as a marker of M2 phenotype was assessed by multi-parameter flow cytometry [47]. The employed gating strategy is shown in Fig. 2C. Results showed a significant difference in the expression of surface markers between normal MΦ and TAMs (Fig. 2D–F). M2 phenotype was more prevalent in the case of TAMs as evidenced from the low median fluorescence intensity (MFI) of CD40 and CD86 and high MFI of CD206 compared to MΦ. Moreover, it was evident that, in the case of CD86 and CD206, two discrete negative and positive populations of cells exist; however, Mel secretome seemed to only affect the level of expression, but not the percentage of positive cells. Upon exposure of TAMs to M-NPs, an increased expression of CD40 was observed (Fig. 2D). Similarly, both the MFI and percentage of CD86 positive cells were significantly heightened (Fig. 2E) in contrast to the changes observed in the expression of CD206 (Fig. 2F).

#### Mel secretome mediated a transformation of Fbls to a more aggressive phenotype, the TAFs, while L-NPs restored the naïve phenotype

**Fig. 3:**
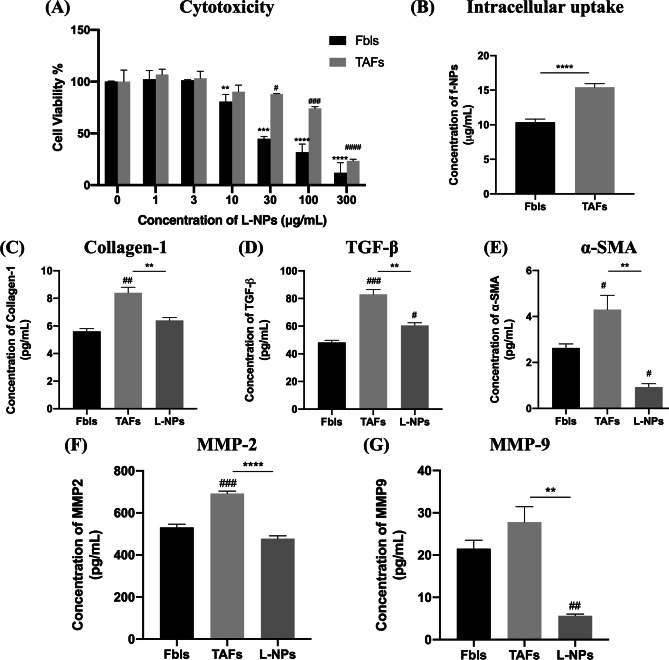
Impact of L-NPs on tumor-associated fibroblasts (TAFs). **A** Cytotoxicity of L-NPs in normal Fbls and TAFs. **B** Intracellular uptake of f-NPs in normal Fbls and TAFs. **C**–**G** mRNA expression of collagen-1, TGF-β, α-SMA, and MMP-2 and 9 respectively in normal Fbls, TAFs, and TAFs treated with IC_50_ (169.8 µg/mL) of L-NPs quantified by RT-q-PCR. Statistical analyses were performed by *t*-test or one-way ANOVA using GraphPad Prism Software 8.3. In insert **A**, means of treated cells were normalized to the mean of control cells. * denotes comparison in the case of Fbls, and ^#^ denotes comparison in the case of TAFs. In inserts **C**–**G**, * denotes comparison to TAFs, and ^#^ denotes comparison to Fbls. Levels of significance are indicated as follows: */^#^*P* < 0.05; **/^##^*P* < 0.01; ***/^###^*P* < 0.001; ****/^####^*P* < 0.0001

Mel secretome affected Fbls in a similar manner to how it affected MΦ, mediating their transformation into a more aggressive phenotype, the TAFs. L-NPs have shown significant toxicity to Fbls with an IC_50_ of 35.9 ± 4.0 µg/mL (Fig. 3A). However, upon pre-incubation of Fbls with Mel-conditioned medium to generate TAFs, L-NPs were shown to lose their potency recording an IC_50_ of 169.8 ± 19.5 µg/mL. To evaluate whether this is due to a lower tendency of the accumulation of the NPs in TAFs or not, the intracellular concentration of f-NPs was assessed (Fig. 3B). Contrarily to what was expected, a higher accumulation of the f-NPs was recorded in TAFs than Fbls. Upon evaluation of TAF-related markers, an overexpression of collagen 1, TGF-β, α-SMA, and MMP-2 and 9 was observed in TAFs relative to Fbls (Fig. 3C–G respectively).

Fortunately, L-NPs treatment could significantly downregulate collagen-1 and MMP-2 to baseline levels of normal Fbls (Fig. 3C, F respectively), while they could significantly ablate α-SMA and MMP-9 to lower-than-normal levels (Fig. 3E, G respectively). In the case of TGF-β expression, L-NPs could as well trigger a significant downregulation when compared to TGF-β levels in TAFs; however, levels were still higher than normal Fbls (Fig. 3D).

#### The secretome of untreated and treated TAFs and TAMs impact the efficacy of D-NPs in B16F10 melanoma cells differently

**Fig. 4:**
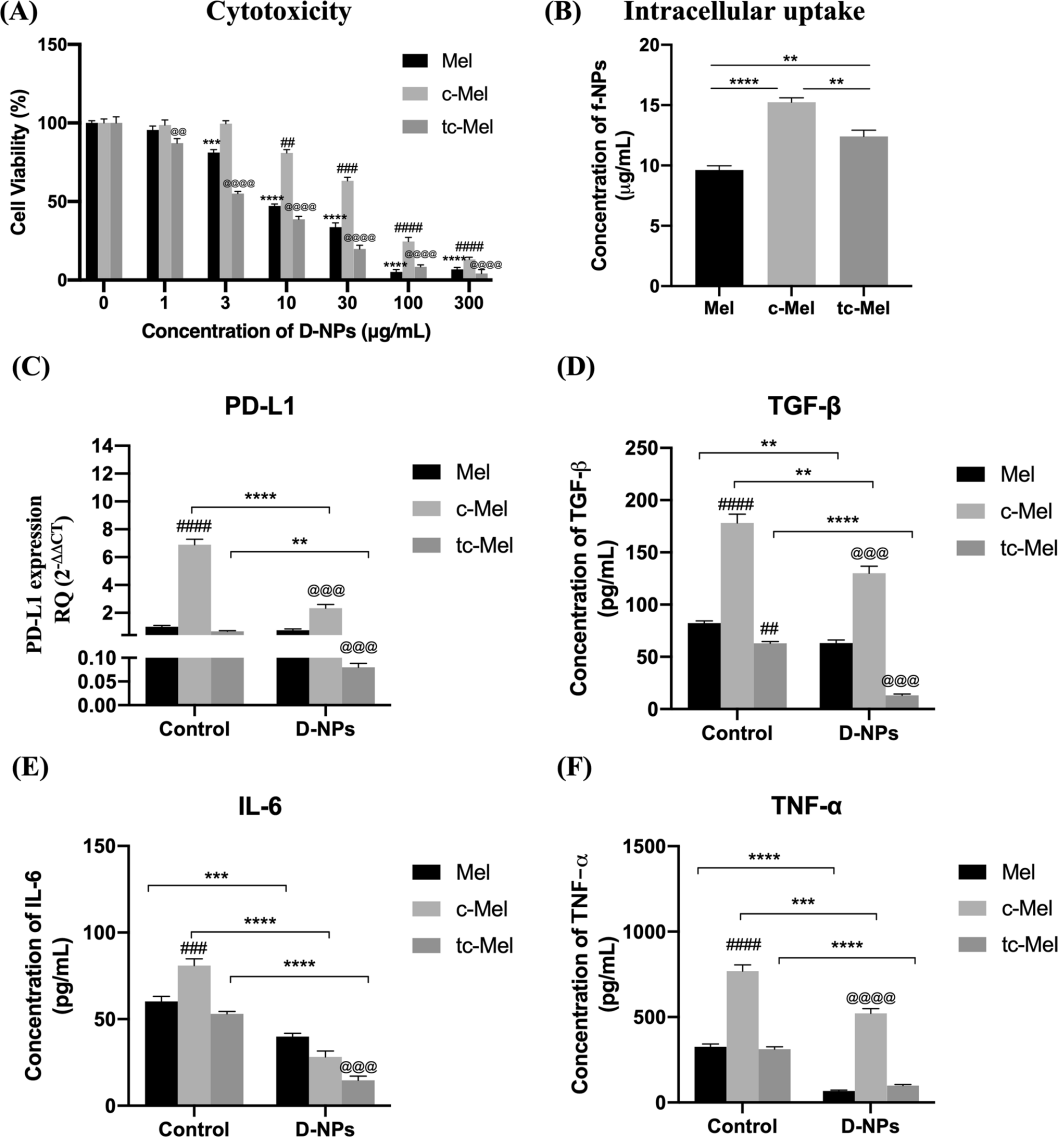
Impact of D-NPs on B16F10 melanoma cells. **A** Cytotoxicity of D-NPs in normal Mel, Mel pre-incubated with TAFs and TAM-conditioned medium (c-Mel), and Mel pre-incubated with treated TAF and TAM-conditioned medium (tc-Mel). **B** Intracellular uptake of f-NPs in Mel, c-Mel, and tc-Mel. **C**–**F** mRNA expression of PD-L1, TGF-β, IL-6, and TNF-α respectively in Mel, c-Mel, and tc-Mel treated with IC_50_ (11.4, 43.8, and 5.3 µg/mL respectively) of D-NPs quantified by RT-q-PCR. Statistical analyses were performed by one- or two-way ANOVA using GraphPad Prism Software 8.3. In insert **A**, means of treated cells were normalized to the mean of control cells. * denotes comparison in the case of Mel, ^#^ denotes comparison in the case of c-Mel, and ^@^ denotes comparison in the case of tc-Mel. In inserts **C**–**F**, ^#^ denotes comparison of control c-Mel and tc-Mel to control Mel, ^@^ denotes comparison of D-NPs-treated c-Mel and tc-Mel to D-NPs-treated Mel. * denotes comparison between control and treated cells. Levels of significance are indicated as follows: */^#^/^@^*P* < 0.05; **/^##^/^@@^*P* < 0.01; ***/^###^/^@@@^*P* < 0.001; ****/^####^/^@@@@^*P* < 0.0001

The anti-tumor efficacy of the D-NPs was evaluated in monotherapy by direct application on normal Mel and Mel pre-incubated with conditioned media derived from cultures of both TAMs and TAFs (c-Mel) to investigate whether the secretome of TME components would impart anti-cancer therapy resistance or not. Moreover, D-NPs were evaluated in combination with TME modulatory NPs (M-NPs and L-NPs) by applying D-NPs on Mel pre-incubated with conditioned medium derived from cultures of TAMs and TAFs treated with IC_50_ concentrations of M-NPs and L-NPs respectively (tc-Mel). Results showed IC_50_ of 11.4 ± 0.9, 43.8 ± 3.2, and 5.3 ± 0.5 µg/mL in Mel, c-Mel, and tc-Mel respectively (Fig. 4A). The decrease in potency of D-NPs in c-Mel relative to Mel could not be attributed to the intracellular concentration of f-NPs as f-NPs showed significantly higher cellular uptake in c-Mel over Mel (Fig. 4B). Needless to say, the expression of all markers in c-Mel was powerfully boosted compared to normal Mel (Fig. 4C–F). Contrarily, the expression of markers in tc-Mel was either unaffected or slightly downregulated as in the case of TGF-β upon comparison to Mel (Fig. 4D). D-NPs monotherapy in Mel did not affect PD-L1 level but could significantly downregulate the mRNA expression of TGF-β, IL-6, and TNF-α (Fig. 4D–F respectively). The impact of D-NPs monotherapy on c-Mel was stronger in which the expression of all the markers was significantly dampened. D-NPs combination with conditioned media from M-NP-treated TAMs and L-NP-treated TAFs rather produced the most powerful repression of all the immunosuppressive markers.

### Metformin and losartan heightened the tumor and spleen accumulation of NPs

**Fig. 5:**
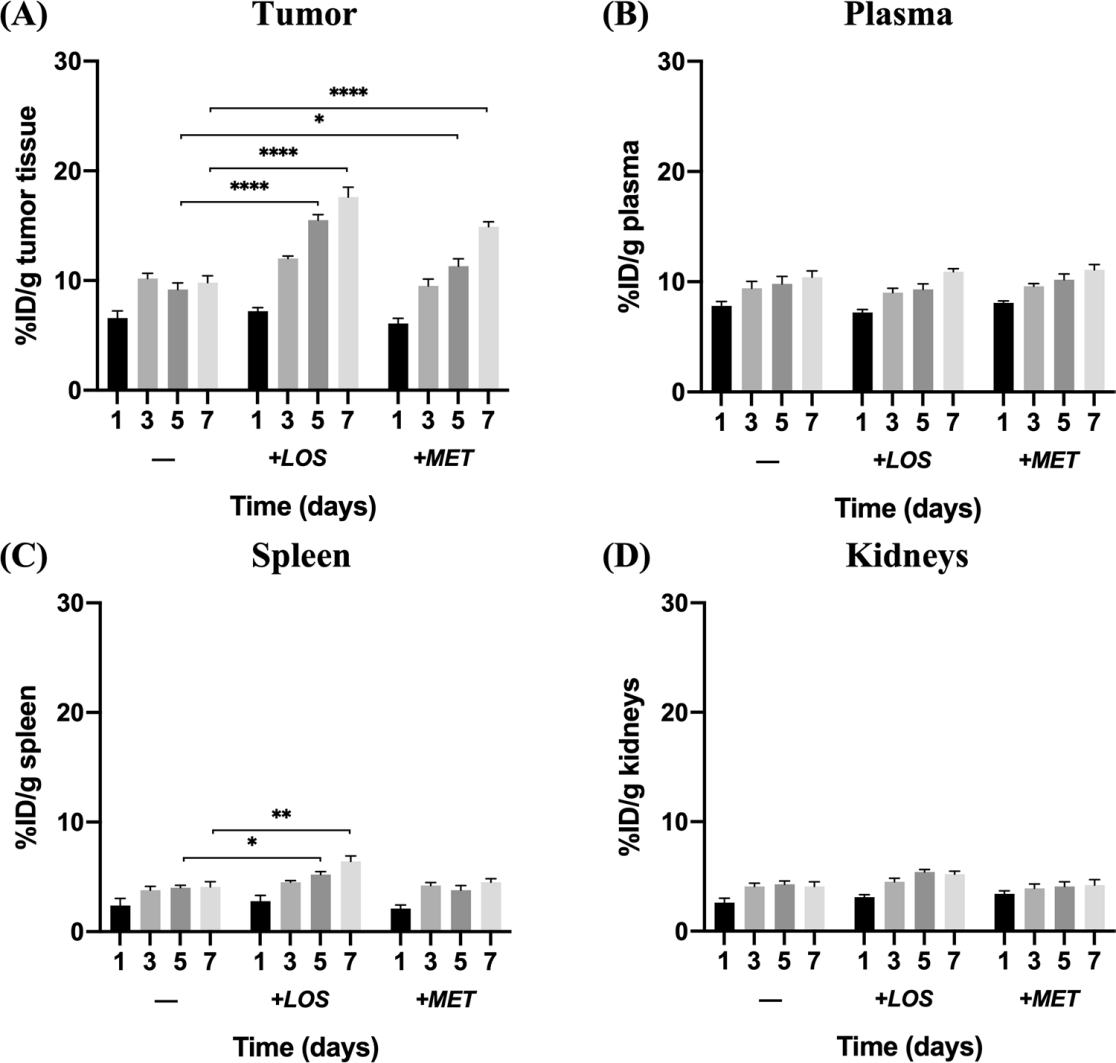
Biodistribution of f-NPs, f-L-NPs, and f-M-NPs in melanoma-bearing C57/Bl6 mice. Accumulation of NPs in tumor tissues (**A**), plasma (**B**), spleen (**C**), and kidneys (**D**). Statistical analysis was performed by two-way ANOVA using GraphPad Prism Software 8.3. Levels of significance are indicated as follows: *P < 0.05, **P < 0.01, ****P < 0.0001

PLGA f-NPs have shown a high accumulation in the tumor tissue and plasma (Fig. 5A, B) that slightly increased over time upon successive administration, while NPs’ accumulation in the spleen and kidneys occurred to a lower extent (Fig. 5C, D). Upon co-loading the NPs with losartan, the tumor and spleen accumulation increased significantly starting at day 5 while no effect was observed in the plasma or kidney levels. Metformin as well could increase the tumor accumulation of the NPs but to a lesser extent. However, no change in the levels of NPs in plasma, spleen, or kidneys were observed.

### In vivo anti-tumor efficacy of mono- and combination therapies

#### D-NPs in mono- and combination therapies produced a cascade of local anti-tumor and immunomodulatory effects

**Fig. 6:**
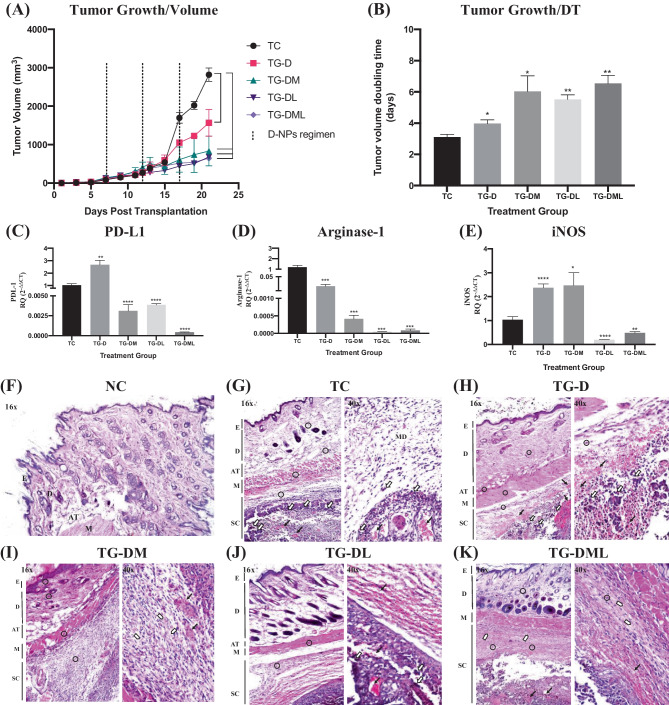
In vivo local anti-tumor efficacy of mono- and combination therapies. **A** Tumor growth curves expressed in terms of volume (mm^3^) recorded over a 21-day duration post melanoma cell transplantation. **B** Tumor volume doubling time (DT) in days calculated by nonlinear fit of growth curves “Exponential (Malthusian) growth” using GraphPad Prism software 8.3. **C**–**E** mRNA expression of PD-L1, arginase-1, and iNOS in the tumor tissue quantified by RT-qPCR respectively. **F**–**K** Histopathological evaluation of normal skin from NC and tumor-bearing skin from TC, TG-D, TG-DM, TG-DL, and TG-DML respectively. Symbols and labels in the histopathological images indicate the following: E, epidermis; D, dermis; AT, adipose tissue; M, muscles; SC, subcutaneous; MD, myxomatous degeneration. 〇, inflammatory cells;  , fibrosis; ⇩, melanoma cells; ↓, necrosis. Statistical analyses in inserts **B**–**E** were performed by one-way ANOVA by comparing the mean of each group to the mean of TC using GraphPad Prism Software 8.3. Levels of significance are indicated as follows: *P < 0.05, **P < 0.01, ***P < 0.001, ****P < 0.0001

The impact of monotherapy of D-NPs or combination with L-NPs and/or M-NPs on the local microenvironment of melanoma has been assessed. The growth rate of the tumors in the TC and TGs has shown an exponential pattern **(**Fig. 6A). Upon comparison of the tumor volumes of different TGs, a slower growth of tumor was observed in the case of mono- and combination therapies. This was further proven upon calculation of the DT (Fig. 6B), whereas a significantly prolonged DT of the tumor volume was observed in the TGs. The combination therapy possessed an advantage with regard to tumor growth rates as evidenced by the lower tumor volume at day 21 post-transplantation and the calculated DT. Upon molecular analyses of markers of immunosuppression, a significant downregulation of PD-L1 was observed only in the combination therapies as compared to D-NPs alone (Fig. 6C). Surprisingly, D-NPs alone significantly increased the PD-L1 level in the tumor tissue. Moving to the expression levels of arginase-1, mono- and combination therapies could effectively downregulate its expression (Fig. 6D). In the case of iNOS levels, a significant upregulation was observed in TG-D and TG-DM, while losartan-containing regimens produced an opposite effect (Fig. 6E).

Histological analyses of NC revealed the normal structure of the epidermis and dermis with the underlying subcutaneous (SC) adipose tissue (Fig. 6F). In the TC, melanoma cells were observed as dark purple spots in the SC tissue (Fig. 6G). Inflammatory cells were detected in a diffuse manner in the dermis, SC tissue, and in between muscle bundles. Myxomatous degeneration (MD) and edema were noted in the SC tissue surrounding melanoma cells. In TG-D, melanoma cells were evident in the SC layer. While the epidermal and dermal tissue layers showed intact histological structure associated with infiltration of few inflammatory cells and focal hemorrhage (Fig. 6H). Upon combining D-NPs with M-NPs, the most pronounced changes were the appearance of necrotic regions and fibrotic areas (Fig. 6I). The infiltration of inflammatory cells was observed to be more intense as well. The epidermal and dermal layers of the skin in TG-DL resembled the TG-D showing intact histological structure associated with few inflammatory cells’ infiltration and focal hemorrhages (Fig. 6J). The absence of fibroblastic cells was evident in this group. In the last group, TG-DML, where the three therapeutic agents were combined, inflammatory cells were observed in both of the dermis and SC tissue. In addition, the SC tissue showed inflammatory cell infiltration, fibroblastic cell proliferation, and focal hemorrhages (Fig. 6K).

#### D-NPs in mono- and two-agent combination therapy produced desirable systemic effect, while the three-agent combo mediated a status of systemic immune depletion

**Fig. 7:**
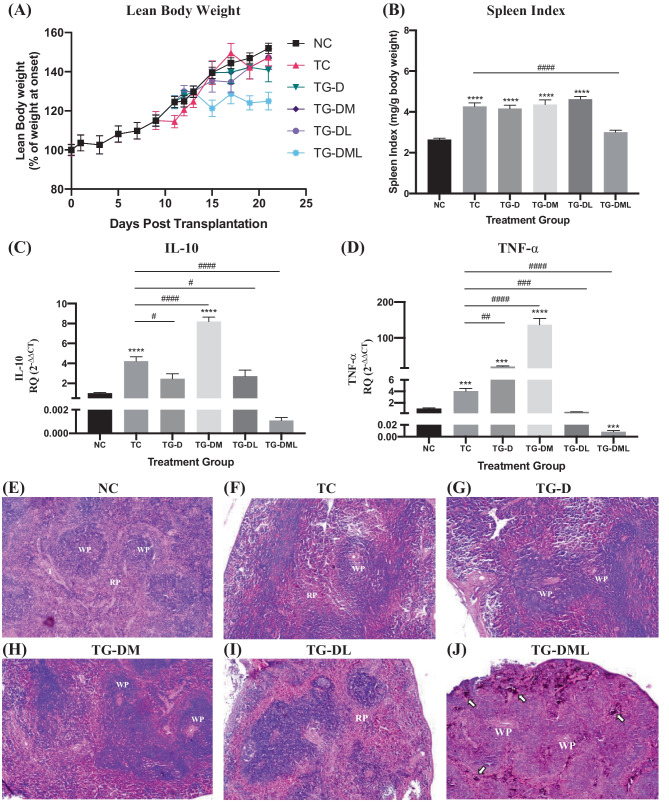
In vivo systemic immune responses of mono- and combination therapies. **A** Lean body weight expressed as percentage of weight at onset (%). **B** Spleen index expressed as weight of spleen relative to the weight of mouse after euthanasia (mg/g) in the different treatment groups. **C** and **D** mRNA expression of IL-10 and TNF-α in the spleen quantified by RT-qPCR respectively. **E**–**J** Histopathological evaluation of spleen from NC, TC, TG-D, TG-DM, TG-DL, and TG-DML respectively. Symbols and labels in the histopathological images indicate the following: WP, white pulp; RP, red pulp; and ⇩, melanoma cells. Statistical analyses in inserts **B**–**D** were performed by one-way ANOVA using GraphPad Prism Software 8.3. * denotes analysis performed by comparing the mean of each group to the mean of NC. ^#^ denotes analysis performed by comparing the mean of each group to the mean of TC. Levels of significance are indicated as follows: */^#^*P* < 0.05; **/^##^*P* < 0.01; ***/^###^*P* < 0.001; ****/^####^*P* < 0.0001

Lean body weight of mice over the course of treatment was evaluated as an indicator of the overall well-being of mice [48]. Mice in all the groups maintained a similar growth in body weight to the NC group except for the TG-DML in which a significant decrease in the body weight was observed starting from day 13 post-transplantation (Fig. 7A). Moreover, spleen index was calculated as an indicator of development of an immune response or the status of immune depletion [43]. Mice in TC, TG-D, TG-DM, and TG-DL exhibited a significantly higher spleen index than mice in NC group (Fig. 7B). However, a drop in the spleen index was observed in the TG-DML. In order to gain a deeper insight into the immune system status, the expression of IL-10 and TNF-α in the spleen was evaluated in different groups (Fig. 7C, D respectively). IL-10 expression was significantly elevated in the TC group as compared to the NC. However, a significant downregulation was observed upon treatment with D-NPs alone or in the L-NP- containing combination regimens. Surprisingly, the expression of IL-10 in the TG-DM was significantly upregulated to exceed even the TC levels (Fig. 7C). Similar pattern of expression of TNF-α was observed except that in the spleen of mice receiving the monotherapy of D-NPs, an upregulation was observed (Fig. 7D). Further to the spleen index evaluation and inflammatory markers expression analyses, histopathological assessment of spleens from different groups was performed. Normal histopathological structure of the white and red pulps and connective tissue trabeculae was observed in the spleen of NC mice (Fig. 7E). In the spleens of mice in TC, TG-D, and TG-DM, lymphoid hyperplasia in the follicles of the white pulps was observed (Fig. 7F–H). Contrarily, a slight lymphoid depletion in the white pulps was observed in the spleen of TG-DL mice (Fig. 7I). This was more evident in the TG-DML spleen accompanied by an accumulation of deep brown-to-black melanoma cells (Fig. 7J).

## Discussion

TME remodeling strategies are receiving increasing interest for their potential to enhance the treatment outcomes of different anti-cancer agents including chemotherapy and immunotherapy. Among different TME components, TAMs and TAFs contribute to a great extent to the failure of obtaining an adequate outcome by creating a drug-resistant and immunosuppressive environment. Efforts have been spent over the past decade to repolarize TAMs and TAFs into their naïve or anti-tumorigenic phenotypes. In this context, drug repurposing has flourished to a great extent. The aim of this study was to evaluate the efficacy of doxorubicin as an ICD agent upon combination with TME remodeling agents in an engineered nanoparticle system for maximizing treatment benefit and minimizing side effects.

PLGA NPs with suitable properties for tumor targeting by the EPR effect were successfully obtained. NPs with an average diameter of approximately 150 nm are known to preferentially accumulate in the tumor tissue [37]. PDI < 0.3 indicates that PLGA NPs are sufficiently monodispersed and ZP around −20 mV points to acceptable standing stability [1, 15]. The EE% results as well are in accordance with previous reports where PLGA NPs obtained by double emulsion solvent evaporation method exhibited high EE of different drugs including those with high molecular weight [49]. Moreover, spherical particle shape and smooth surface morphology are other characteristic properties of PLGA NPs [1].

On the in vitro level, TAMs and TAFs were successfully established from MΦ and Fbls respectively via incubation with Mel secretome. This protocol is currently more widely utilized over the use of specific polarizing or stimulating agents as means to approach the real exposure in the TME [12, 33]. The successful establishment of TAMs is clear from the increased expression of CD206 (M2 marker) and decreased expression of CD40 and CD86 (M1 markers) compared to MΦ. Similar findings were observed for macrophages or monocytes incubated with conditioned media derived from cultures of colon [50] and breast [51] cancer cells. Similarly, Mel secretome-incubated Fbls showed TAF-polarized phenotype. TAFs are characterized by higher contractility with an elevated expression of α-SMA, MMPs, and ECM proteins, e.g., type I collagen. Moreover, TAFs secrete higher amounts of different growth factors and TME mediators including TGF-β to promote tumorigenesis, invasiveness, and metastasis [6, 52]. Similar results were observed in myofibroblasts treated with the secretome of metastatic colon cancer cells which showed increased α-SMA and MMPs expression and collagen deposition [53].

To elaborate on the role of M-NPs in MΦ and TAMs, cytotoxicity analysis was performed. The reduced cytotoxic effect of M-NPs in TAMs indicates a possible decrease in the phagocytic potential of MΦ upon exposure to Mel secretome as indicated from the intracellular uptake experiments. In previous studies, TAMs were shown to exhibit a slower endocytic uptake mechanism than normal MΦ; TAMs rely on a slow macropinocytosis process unlike MΦ which follow an ultrafast clathrin-mediated endocytosis [54, 55]. Moreover, this decrease in the cytotoxicity of M-NPs could also point to the development of a resistance mechanism in TAMs or to a phenotype-dependency of the cytotoxic effect as shown before [56]. Despite the lower cytotoxic effect, M-NPs were able to skew TAMs from M2 to M1 phenotype in consistency with previous reports [57, 58]. This shift in polarity may be due to the metformin-induced increase of intracellular oxygen concentrations as a general reduction in hypoxia shifts TAM polarity towards M1 [19].

Regarding the relative efficacy of L-NPs in Fbls and TAFs, the reduced sensitivity of TAFs to L-NPs compared to normal Fbls harmonizes with previous studies that showed a potential role of the overly expressed TGF-β in the development of therapy resistance despite the higher accumulation of NPs which is another distinct feature of TAFs [59, 60]. The ability of L-NPs to transform TAFs to quiescence stems from losartan’s ability to downregulate TGF-β through NFкB inhibition [61]. This brings about a cascade of inhibitory effects on other TAF distinct features including collagen-1 and MMP expression [62].

Results of cytotoxicity experiments of D-NPs on B16F10 cells indicated effective monotherapy in the case of Mel, development of anti-cancer therapy resistance in the case of c-Mel, and efficacy of the combination therapy in the case of tc-Mel. Doxorubicin has well been utilized as a potent chemo(-immuno) therapeutic agent; however, its impact on PD-L1 has been subject to debate. Some studies established that it possesses an inhibitory impact on PD-L1 via mediating mRNA degradation [63], while others have shown that doxorubicin, through inhibition of miR-140 expression, upregulates PD-L1 expression in a cancer-type-dependent manner [64]. Another evidence of this PD-L1 up-regulatory effect of doxorubicin has been provided by Ghebeh et al. (2010) who showed that doxorubicin-dependent cell surface downregulation of PD-L1 was accompanied by its upregulation in the nucleus [65]. This, at least in part, explains the discrepancy observed between the in vitro downregulation and in vivo upregulation of PD-L1 in TG-D.

The upregulation of PD-L1, TGF-β, IL-6, and TNF-α observed in c-Mel represents further evidence on the immunosuppressive roles of TAFs and TAMs on cancer cells. Previous studies pointed to an up-regulatory effect of M2 TAMs and TAFs on the expression of IL-10 and TGF-β [66, 67], IL-6 and MMPs [12, 68], and the endothelial mesenchymal transition (EMT)-related genes, e.g., vimentin [69] in cancer cells which subsequently dampened the efficacy of ICD.

The efficacy of the combination therapy could, however, be attributed to the altered secretome of re-educated TAMs and silenced TAFs or to the presence of dissolved metformin and losartan in the respective conditioned medium or both. According to the release experiment shown in Fig. S2, 32.2% of metformin is released from M-NPs over 24 h corresponding to a concentration of less than 5.5 µg/mL. Similarly, 23.4% of losartan is released from L-NPs resulting in a concentration of approximately 10 µg/mL in the conditioned medium. Both losartan and metformin were previously reported to alleviate the immune checkpoint expression through reduction of TGF-β [62] and IL-6 [70] respectively.

In the melanoma-bearing mouse model, the effect of metformin and losartan was first witnessed on the tumor accumulation of NPs. PLGA f-NPs could benefit from the EPR effect by virtue of possessing physicochemical properties suitable for tumor targeting [37]. However, TME priming by metformin or losartan could enhance the tumor penetration by virtue of the ability of both agents to induce stromal depletion as evidenced earlier in pancreatic cancer in the case of metformin [71] and breast, pancreatic and skin cancers in the case of losartan [63].

Following the biodistribution studies, it was necessary to evaluate the tumor burden in the TC and the responses to therapeutic agents in the TGs. In TC, it was clear that the body responds to the tumor burden drastically both locally and systemically via the release of different immunosuppressive cytokines and markers such as IL-10, TNF-α, and iNOS which further support the tumor progression [72, 73]. In addition, observation of MD in TC indicates a progressive loss of the skin integrity due to the alteration in the architecture and composition of the tissue [74]. Moreover, the splenomegaly indicated from the elevated spleen index indicates that melanoma growth resulted in a striking shift in the composition of the immune system [75].

D-NPs induced a cascade of local and systemic alterations in the TG-D ranging from reduction in tumor growth, alleviation of the immunosuppressive nature in the TME, and activation of the immune system. These effects have been previously reported for doxorubicin in colon, breast, and skin cancers [76, 77]. However, doxorubicin monotherapy of solid tumors has also been reported to be insufficient [14].

Upon evaluation of the efficacy of the combination therapies, M-NP addition to the treatment program produced a strong elevation in the levels of IL-10 and TNF-α indicating activation of a powerful systemic anti-tumor immune response [48]. Despite that these cytokines mediate opposite effects; their parallel overexpression was interpreted as an indicator of a high number of tumor-infiltrating lymphocytes (TIL) in treated melanoma-bearing mice [78]. The anti-tumor immunostimulatory effects of metformin were further emphasized by a decrease in the levels of PD-L1 and Arginase-1 as previously observed in breast [19] and colon [79] cancers. Moreover, the observed signs of fibrosis in the skin histopathology could be regarded as a desirable outcome of the treatment program that can limit tumor growth and progression [80].

The combination therapy in TG-DL, however, has raised some doubts on the effect of losartan. In the view of tumor growth only, losartan could be regarded as a potential candidate for priming the TME to increase the efficacy of ICD as previously indicated in breast cancer [48, 81]. However, the lower levels of Arginase-1, iNOS, and IL-10 along with the lymphoid depletion observed in the spleen histopathology point to a status of immune depletion in the treated mice. The discrepancy in the results observed in the current study compared to the previous report on the positive impact of combining losartan and liposomal doxorubicin [48] could be attributed to either the cancer type or the effective dose of losartan. In the previous study, the authors used losartan in its free form at a dose of approximately 0.8 mg/day per mouse over 5 days. While in the current study, a dose of approximately 0.2 mg/day per mouse has been utilized in a nano-based system over 14 days. This is not surprising as previous reports showed that a low dose of NP-loaded drug was more effective than a high dose of the drug in the free form possibly due to the higher tumor accumulation and lower clearance [82]. These deleterious effects support the claim that TAF elimination comes with a risk of an increase in tumor progression and metastasis [83].

The adverse effects of combination therapies were striking in the three-agent combo in the TG-DML in which the decrease in systemic inflammation, the lymphoid depletion in the spleen, and the changes in the skin architecture were most evident. Moreover, evidence of metastasis in the spleen was also recorded. In addition, signs of therapeutic toxicity were observed in the form of a decrease in the spleen index and the lean body weight of treated mice [43, 48]. These findings go in line with previous reports showing a relationship between excessive ECM depletion and intratumoral hemorrhage, invasion and metastasis [84–86]. From these findings, the TME has been concluded as a highly orchestrated system, in which modulation of one aspect can bring about unfavorable adaptation of other aspects [6].

## Conclusion

In this study, TME remodeling has been employed as means to counteract the limited efficacy of chemoimmunotherapy and provide a potential therapeutic program for melanoma treatment. On the in vitro level, the TME remodeling agents have shown great efficacy in their target cells: L-NPs in TAFs and M-NPs in TAMs. Moreover, the combination of the secretome of remodeled TAFs or TAMs with D-NPs has shown great promise in dampening different aspects of the immunosuppressive nature of melanoma cells. To the best of our knowledge, the proposed combination therapy was not previously tested in preclinical or clinical studies. We could observe from the in vivo investigations that losartan and metformin, at doses that did not affect the tumor growth, could significantly enhance the tumor accumulation of NPs. Moreover, the combination of either agent with D-NPs has shown some advantages, especially in the case of M-NPs. While L-NP-containing regimens produced alarming side effects that ring a bell at the necessity of employing a dose-reduction strategy. We anticipate reducing the dose of D-NPs in L-NP-containing regimens in future work. In general, anti-cancer combination therapies must be treated with caution. Since the TME is a highly regulated system, its aggressive engineering is a double-edged sword, which might fire back if not properly controlled.

### Supplementary Information

Below is the link to the electronic supplementary material.

Supplementary file1 (DOCX 110 KB)

## Data Availability

The authors confirm that the data supporting the findings of this study are available within the article and the supplementary materials.
