# Supplementary material for: Multitarget, multiagent PLGA nanoparticles for simultaneous tumor eradication and TME remodeling in a melanoma mouse model
Source: Drug Deliv Transl Res. 2023 Aug 23;14(2):491–509. doi: 10.1007/s13346-023-01413-9 (PMC10761550; doi:10.1007/s13346-023-01413-9)
Supplement: Supplementary file 1 — Supplementary file1 (DOCX 110 KB) [file 13346_2023_1413_MOESM1_ESM.docx]

**Multitarget, Multiagent PLGA Nanoparticles for Simultaneous Tumor Eradication and TME Remodeling in a Melanoma Mouse Model**

Asmaa Ramzy^1†^, Aya Soliman^2†^, Sally I. Hassanein^3^, Aya A. Sebak^1*^

^1^ Department of Pharmaceutical Technology, Faculty of Pharmacy & Biotechnology, the German University in Cairo, New Cairo 11511, Egypt

^2^ Department of Pharmaceutical Biology, Faculty of Pharmacy & Biotechnology, the German University in Cairo, New Cairo 11511, Egypt

^3^ Department of Biochemistry, Faculty of Pharmacy & Biotechnology, the German University in Cairo, New Cairo 11511, Egypt

* Correspondence should be addressed to Aya A. Sebak; [aya.sebak@gmail.com](mailto:aya.sebak@gmail.com).

^†^ These authors contributed equally

# **Methods**

## ***Determination of the yield of fluorescein loaded or co-loaded NPs***

The washed pellet of the nanoparticles (NPs) was freeze-dried in FDU 2100 (EYELA, Japan) for 48 h without any additives, then weighed. The yield (%) was then calculated according to the following equation;

$Yield= \frac{Actual weight}{Added weight of PLGA+added weight of fluorescein+added weight of the drug} x 100$ **(Equation S1)**

## ***Determination of limits-of-detection (LODs) of doxorubicin, losartan and metformin***

Solutions of doxorubicin (5-50 µg/mL), losartan (1-128 µg/mL) and metformin (2-12 µg/mL) were prepared in phosphate-buffered saline (PBS), pH 7.4 and analyzed spectrophotometrically in triplicates at wavelengths of 480 nm, 210 nm and 231 nm respectively using a double beam spectrophotometer (JASCO V- 630, Japan). LODs were calculated according to the following equation;

$LOD= \frac{3.3 \sigma}{S}$ **(Equation S2)**

in which σ is the standard deviation of the intercept and S is the slope of the calibration curve.

## ***Evaluation of the in vitro release of doxorubicin, losartan and metformin from NPs***

A volume of NP suspension containing 0.5 mg of each drug was placed in a Dialysis Tubing Cellulose Membrane of 14,000 Da molecular weight cut-off (Sigma-Aldrich, Germany). The tubing was then tightly sealed and immersed in 5 mL PBS in a closed vessel. Afterwards, the vessel was placed in a shaking water bath adjusted at 100 rpm and 37 °C. Samples of 0.5 mL were withdrawn after 1, 2, 4, 8 and 24 h, replenished with fresh buffer incubated at 37 °C, and analyzed spectrophotometrically for the determination of the concentration of released drugs ^1^.

## ***Biodistribution detailed calculations***

- First, the fluorescence intensity was measured for the diluted organ, issue homogenates or plasma (at a concentration of 0.01g/mL of NaOH) of the treated (NPs-injected) or the control (saline-injected) groups. The weight of the plasma before dilution was calculated from the volume based on a specific gravity of 1.021 g/mL.
- Values were then substituted into the relevant calibration curves constructed in similarly diluted organ, tissue homogenates or plasma (0.01g/mL) of the control group to obtain the diluted concentration of NPs (mg/mL).
- Amounts of NPs (mg) were then calculated taking into consideration both the dilution factor and the homogenate volume.
- The amounts of NPs in the treated (real) and the control (hypothetical; based on possible autofluorescence) were then normalized to the organ, tissue or plasma weights. Normalized amounts of NPs were then expressed as mg/g organ weight.
- Amounts of NPs per gram of organ weight of control mice were then subtracted from the corresponding values from the treated mice.
- Finally, the normalized amounts of NPs were divided by the injected dose and expressed as a percentage of injected dose per organ weight (%ID/g).

# **Results and discussion**

## ***Yields of fluorescein loaded or co-loaded NPs***

The yield (%) of fluorescein-loaded NPs (f-NPs) was slightly higher than fluorescein co-loaded metformin or losartan NPs (f-M-NPs or f-L-NPs respectively) **(Table S1)**.

**Table S1**: Yields (%) of fluorescein loaded or co-loaded NPs

| **NPs type** | **Yield (%)** |
| --- | --- |
| f-NPs | 86.7 ± 4.8 |
| f-M-NPs | 92.1 ± 6.8 |
| f-L-NPs | 91.1 ± 4.7 |

## ***Limits-of-detection (LODs) of doxorubicin, losartan and metformin***

A linear relationship was observed between the concentration of doxorubicin, losartan or metformin and the absorbance, recording LODs of 1.3, 2.0 and 0.4 µg/mL respectively **(Figure S1 A-C)**.

**A**

**B**

**C**

Figure S1: **Calibration curves** of Doxorubicin (A), Losartan (B) and Metformin (C) in PBS exhibiting limit-of-detection (LOD) of 1.3, 2.0 and 0.4 µg/mL, respectively.

## ***In vitro release of doxorubicin, losartan and metformin from NPs***

Metformin showed the highest cumulative released % over 24 h of around 32% despite having the lowest water solubility (33 g/L, Selleckchem, USA) owing to its small molecular weight (165.62 g/mol). Doxorubicin and losartan which are characterized by similar water solubility (100 and 92 g/L) and molecular weight (579.98 and 462.01 g/mol) respectively showed similar cumulative released % of around 25% after 24 h. These results are in accordance with previous reports showing an inverse relationship between the *in vitro* drug release and the molecular weight ^2^ and a direct relationship with the drugs’ water solubility ^3,4^.


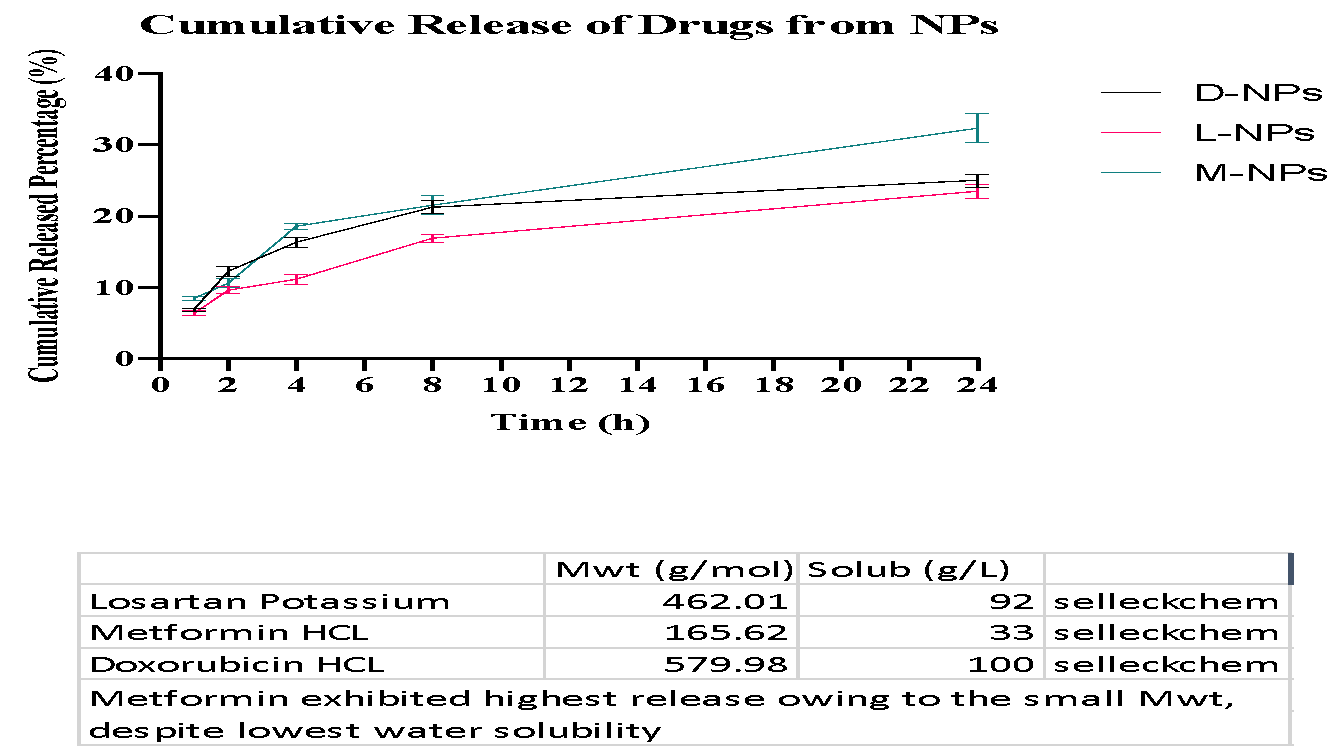


*Figure S2:* ***In vitro release of drugs from PLGA NPs.*** *Cumulative released percentage of doxorubicin, losartan and metformin from D-NPs, L-NPs and M-NPs respectively over 24 h in PBS pH 7.4 at 37 ºC.*

## ***The effect of f-NPs, f-M-NPs or f-L-NPs on tumor growth***

The growth curves of the tumor-bearing mice that were intravenously administered 100 μL of saline, f-NPs, f-M-NPs or f-L-NPs at a concentration of 30 mg/mL in saline once daily exhibited the same patterns. This volume of f-M-NPs and f-L-NPs contains approximately 0.25 mg of metformin or losartan respectively (equivalent to 10 mg/kg of either drug). This indicated that f-NPs, f-M-NPs or f-L-NPs exerted no tumor regression impact.

*Figure S3:* ***Tumor growth curves*** *expressed in terms of volume (mm^3^) recorded over a 21-day duration post melanoma cells transplantation in tumor-bearing mice that were intravenously administered 100 μL of saline, f-NPs, f-M-NPs or f-L-NPs at a concentration of 30 mg/mL in saline once daily.*

# **References**

1. Gomaa, I., Sebak, A., Afifi, N. & Abdel-Kader, M. Liposomal delivery of ferrous chlorophyllin: A novel third generation photosensitizer for in vitro PDT of melanoma. *Photodiagnosis Photodyn. Ther.* **18**, 162–170 (2017).

2. Feng, S., Nie, L., Zou, P. & Suo, J. Effects of drug and polymer molecular weight on drug release from PLGA-mPEG microspheres. *J. Appl. Polym. Sci.* **132**, (2015).

3. Li, S. *et al.* Nanomedicine engulfed by macrophages for targeted tumor therapy. *Int. J. Nanomedicine* **11**, 4107–4124 (2016).

4. Joiner, J. B. *et al.* Effects of Drug Physicochemical Properties on In-Situ Forming Implant Polymer Degradation and Drug Release Kinetics. *Pharmaceutics* **14**, 1188 (2022).
